# Caregivers’ perspectives on factors influencing adolescent girls’ engagement in sexual risk-taking in Uganda: A qualitative study

**DOI:** 10.1371/journal.pone.0338724

**Published:** 2025-12-18

**Authors:** Ozge Sensoy Bahar, Fred M. Ssewamala, Flavia Namuwonge, Florence Namuli, Proscovia Nabunya, ICHAD Field Team

**Affiliations:** 1 Silver School of Social Work, New York University, New York City, New York, United States of America; 2 McSilver Institute for Poverty Policy and Research, Silver School of Social Work, New York University, New York City, New York, United States of America; 3 Brown School, Washington University in St. Louis, St. Louis, Missouri, United States of America; 4 International Center for Child Health and Development, Brown School, Washington University in St. Louis, St. Louis, Missouri, United States of America; 5 International Center for Child Health and Development Field Office, Masaka, Uganda; MRC/UVRI Uganda Research Unit On AIDS: MRC/UVRI and LSHTM Uganda Research Unit, UGANDA

## Abstract

Sub-Saharan Africa is home to two-thirds of people living with HIV globally, and women and girls (all ages) account for 62% of all new HIV infections in the region. Sexual risk-taking puts adolescent girls at risk for adverse health outcomes, including HIV/AIDS. Caregivers’ beliefs about adolescent sexual risk-taking have implications for how and when they would discuss this topic with their children. In this study, we conducted semi-structured in-depth interviews with 58 caregivers of adolescent girls to explore their perspectives on factors that influence adolescent girls’ decisions to engage in sexual risk-taking in Uganda. We employed a thematic analysis approach, combining both inductive and deductive methods to analyze the data. A range of protective and risk factors was identified across personal, proximal, and distal contexts. At the personal level, future goals, concerns about negative health consequences, religiosity, temperament, and puberty were identified as contributing factors. At the proximal level, family and peer-related factors were mentioned. At the distal level, poverty and program counseling were discussed. Study results point to the need for combination interventions that include both caregivers and their daughters to reduce sexual risk-taking among adolescent girls in Uganda.

## Introduction

Sexual risk behaviors (SRB) are those actions that increase the likelihood of adverse health outcomes, including the risk of contracting HIV/AIDS [1]. High-risk sexual behaviors such as intercourse without using a condom, unprotected oral/genital contact, early sexual debut, multiple sexual partners, and unprotected anal sex elevate these risks [2]. Research highlights the importance of promoting safe sex practices among children before they reach adulthood. It becomes challenging to change risky sexual behaviors once adolescents develop unhealthy habits [3]. The adverse consequences of unsafe sex are both physical and psychological [4].

Adolescent behavior is shaped by various factors, including family, peers, schools, neighborhoods, and the broader socio-cultural context [5]. Caregivers play a crucial role in safeguarding adolescents from engaging in risky behaviors [6–8]. Moreover, the behavior of the children in a home is directly influenced by the parenting style [9]. Caregivers’ high levels of harsh parenting is positively correlated with increased risky sexual behavior [10], whereas caregivers’ involved and nurturing parenting is inversely associated with these behaviors [11,12]. However, a cross-sectional study with 462 youth in Eswatini did not find any significant association between parenting style and early sexual debut [13]. In light of evolving societal dynamics, traditional families face challenges in providing adequate guidance and supervision due to the impacts of globalization, modernization, and industrialization [14].

Research indicates that family structure significantly influences children’s behavior. A study by Defo and Dimbuene [15] showed the protective role of two-parent families in Cameroon and another study in Uganda [16] documented the positive impact of having resident biological fathers for delayed sexual debut among adolescent girls. Similar results were reported by [17] who used nationally representative data of female and male adolescents (aged 15–17 years) from 12 countries in sub-Saharan Africa and found that adolescents who lived with both parents were more likely to delay sexual debut compared to those living with a single parent or neither parent.

According to the study by El Kazdouh et al. [18], examining risks and protective factors related to risk-taking behaviors can be grouped into five primary themes. At the individual level, risky sexual behaviors were attributed to a lack of sexual education and the increased use of illicit substances such as drugs and alcohol during adolescence. This period is marked by behaviors influenced by a deficient understanding of sexuality. On the relationship level, the study highlighted poor communication between guardians and adolescents, where discussions regarding sexual health were often avoided or seen as disrespectful, potentially leading to an increase in risky behaviors. From a community perspective, schools were recognized for their role in promoting awareness about sexuality and sexually transmitted infections (STIs), although the education provided was deemed inadequate to equip adolescents with the skills to resist temptations. The impact of media was another critical theme, with exposure to pornography being a significant concern. Finally, changes in socio-cultural norms at the societal level were discussed, emphasizing how the normalization of risky behaviors and a lack of comprehensive religious and moral education contribute to adolescents engaging in hazardous activities.

Peer pressure was another factor that was mentioned as a contributing factor by caregivers. For example, a study conducted in Kenya aimed at understanding youth sexual behaviors from the perspective of caregivers found that adolescents often engage in early sexual debut to avoid being perceived as abnormal by their peers. Other studies examining risk factors for sexual risk-taking also identified peer pressure as a critical factor [19,20]. Another factor that caregivers believe contributes to adolescents’ engagement in sexual risk behaviors at the individual level is their impulsive nature, which often leads to poor decision-making and increased vulnerability to risky behaviors [21].

Family sexuality communication offers caregivers a secure environment to address and regulate sexual matters with adolescents [22]. Caregivers play an important role in discouraging unprotected sex [23]. However, caregivers may employ negative language in sex education, deliberately creating frightening images of sexual behavior to emphasize immorality [22]. The fear of promoting promiscuity is cited as a reason why caregivers avoid discussing sex, while adolescents fear being reprimanded, thus keeping sex education in the dark [24].

Caregivers may avoid discussing engagement in SRB with their adolescent children due to their own fear of discussing sexual content with them (adolescents), lack of knowledge, and moralistic and religious views [20,25]. This hesitation leaves adolescents less informed about the risks they may encounter when engaging in certain behaviors [26]. Furthermore, communication about sexual risk behaviors is often hindered in households with a large number of members, as caregivers are more comfortable discussing these topics within smaller families [27]. In addition, studies have found that mothers were likely to communicate about sexual education compared to fathers and girls were more likely to receive sexual education from their caregivers compared to boys [22,20], possibly due to perceptions that girls are more vulnerable to the negative consequences of sexual risk-taking [20].

While previous studies offer valuable insights into the factors influencing young girls’ decisions to engage in SRB, there is a lack of qualitative research from the perspective of caregivers in sub-Saharan Africa, including in Uganda, where HIV rates remain high, with adolescent girls and young women aged 15–24 years being three times more likely to acquire HIV [28]. Understanding caregivers’ perceptions of why adolescent girls engage in sexual risk-taking has implications for developing interventions to strengthen caregiver-adolescent girl communication around sexual health and risk-taking.

## Methods

### Overview of the randomized clinical trial

A 3-arm cluster randomized clinical trial (2017–2023), funded by the National Institute of Mental Health, examined the impact of a combination intervention on reducing HIV risk among adolescent girls who were between 14–17 years old at enrollment (n = 1260) across 47 secondary schools in the Greater Masaka region of Uganda, a region with one of the highest HIV prevalence in Uganda (11.7%) compared to the national average (5.4%) [29]. The *Suubi4Her* combination intervention included a savings-led family economic empowerment component through youth development accounts (YDA) and an evidence-based family strengthening intervention component that was delivered using a multiple family group (MFG) format. The three study conditions were: (1) control condition: bolstered usual care that involved standard health and sex education available at their school and provision of textbooks, notebooks, and pens; (2) treatment condition 1: YDA, Financial Literacy Training (FLT), and income-generating Activities (IGA); and (3) treatment condition 2: YDA-FLT-IGA + MFG. The randomized clinical trial is registered in the clinical trials database (NCT03307226).

The inclusion criteria were: (1) female; (2) ages between 14–17 years; (3) enrolled in the first or second year of secondary school; and (4) living within a family (broadly defined, including kin) (see [30] for more details on recruitment and study conditions).

A qualitative component was included to explore: (a) participants’ and their caregivers’ experiences with the intervention in each treatment condition and perceptions on program sustainability; and (b) caregivers’ perspectives on key multi-level factors that may have impacted participants’ observable behaviors and decision-making concerning savings, mental health, and sexual risk-taking. In this study, we examined caregivers’ perspectives on factors that contribute to adolescent girls’ decision-making on sexual risk-taking.

### Qualitative sampling

We used stratified purposeful sampling to select the adolescent girls for the qualitative component using three key outcomes (sexual risk-taking, mental health, and matched savings) in the two treatment arms. We used the biomarker data for STIs to stratify adolescent girl participants into two groups: those who screened positive (new cases) and negative at 12-month follow-up for sexual risk-taking. We also stratified participants into the lowest and highest quartiles in mental health based on Beck’s Depression Inventory scale [31]. Finally, we stratified adolescent girl participants into the lowest and highest quartiles in savings using YDAs tracked through monthly bank statements. We then randomly selected five adolescent girls and their caregivers from each of the stratified groups across the two treatment arms, resulting in 60 adolescent girls and their caregivers. Fifty-eight caregivers participated in the interviews.

### Data collection

We conducted semi-structured in-depth interviews with caregivers following intervention completion between 25/09/2020 and 25/01/2021 in a private setting at their daughter’s school. To minimize potential discomfort, we started with the following statement: “Sometimes young girls decide to engage in sexual risk behaviors (such as having unprotected sex with a boyfriend, having sex with older men, having sex for money or other goods) for several reasons.” Following this statement, we asked caregivers, “What do you think are the factors that influence young girls’ decision to engage in sexual risk behaviors?”, followed by “How about girls who do not engage in such behaviors? What are the factors that prevent them from doing so?”

All interviews were conducted in person in Luganda, the widely spoken local language in the study region, by seven research assistants fluent in both languages and trained by two authors with qualitative research expertise. The interview guide was translated from English to Luganda and back-translated by team members fluent in both languages. Interview questions were then reviewed by the study team, which included both research assistants and co-investigators fluent in both languages. Necessary revisions were made in the translated version, where necessary, and the interview guide was pre-tested to ensure that the questions sounded natural and conversational, and conveyed the intended meanings. On average, interviews lasted 1 hour and 58 minutes (ranging from 56 minutes to 3 hours and 21 minutes) and were audiotaped.

### Qualitative data analysis

All interviews were transcribed verbatim and translated into English. We used both inductive and deductive techniques for thematic analysis of the data [32,33] in Dedoose analytic software using Eaton et al.’s [34] conceptualization of three contexts that influence sexual risk behavior in sub-Saharan Africa, namely personal (cognitions and feelings about sexual behavior and thoughts about one’s self), proximal (interpersonal relationships and physical and organizational environment), and distal (culture and structural factors). Both sensitizing concepts informed by the existing literature and emergent themes (open coding) were used [35,33]. The themes were then categorized into more specific units until no further subcategories were necessary.

The analysis, conducted by two authors, compared and contrasted themes and categories to identify similarities, differences, and relationships among findings. Additionally, we used data displays to facilitate the conceptualization processes and developed matrix displays for each participant to identify patterns in the data [36]. We used peer debriefing, whereby the codes and the findings were presented to two members of the research team who were not involved in the data analysis to discuss the plausibility of themes and related findings [32,35]. No changes to the themes and findings were deemed necessary.

### Ethical considerations

Participation in the study was voluntary. Written consent was obtained from all participants to participate in the qualitative interviews. Study procedures were approved by the Institutional Review Board at Washington University in St. Louis (IRB #201703102), the Uganda Virus Research Institute (GC/127/17/07/619), and the Uganda National Council for Science and Technology (SS4406). All audio recordings were saved in a password protected computer. In addition, all translated and transcribed interviews were saved in a secure cloud storage using password protected computers, with access to only necessary research personnel. Consent forms were kept in separate and in locked cabinets. Pseudonyms are used in this manuscript to protect participant confidentiality.

## Results

**Participant characteristics.** Of the 58 caregivers who participated in the semi-structured interviews, 74% were female. Fifty-nine percent were mothers, and 17% were fathers. The remaining 24% included aunts, brothers, sisters, stepmothers, grandmothers, and guardians. The mean age for daughters was 16.7 years old at the time of the qualitative interview. The median number of people in the household was seven and the median number of children was three. The average number of household assets was 10.8 (range 0–21). See Table 1 for more details.

**Table 1:** Participant demographics.

Characteristics	Frequency (percentage) or means ± SD (n = 58)
Caregiver gender	
Female	43 (74.1)
Male	15 (25.9)
Caregiver type	
Mother	34 (58.6)
Father	10 (17.2)
Other	14 (24.1)
Daughter’s Age (Range 15–18 years)	16.7 ± 0.88
Household size (Median)	
People (Range 5–9)	7
Children (Range 2–4)	3
Household asset (mean scores)	10.8 ± 3.77

### Qualitative results

Caregivers’ perspectives on factors that influence adolescent girls’ decision to engage in sexual risk-taking were explored within personal, proximal, and distal contexts. Caregivers discussed these factors as a risk or protective factor depending on whether they were present or not in adolescent girls’ lives. Factors at the personal level included future goals, religiosity, one’s temperament, concerns about negative health outcomes, and puberty. Factors at the proximal level included family and peer relations, and factors at the distal level included poverty (see Table 2).

**Table 2:** Themes.

PERSONAL	PROTECTIVE	Future goals Concerns about negative health consequences Religiosity Temperament Puberty
	RISK	Lack of future goals Lack of religiosity Temperament Puberty
PROXIMAL	PROTECTIVE	***Family*** Parental supervision Parental counseling Parents not engaging in sex “in a noticeable way” Positive family relations ***Peers*** Avoiding “bad” peer groups Positive peer role models
	RISK	***Family*** Lack of parental supervision Lack of parental counseling Parents engaging in sex “in a noticeable way” Unhealthy family relations *-Domestic violence/mistreatment* *-Lack of parental warmth* ***Peers*** Negative peer influence
DISTAL	PROTECTIVE	Met material needs Program counseling
	RISK	Unmet material needs Lack of program counseling

### PERSONAL

***Future goals.*** The most frequently identified protective factor at the personal level was adolescent girls’ having future goals. Raymond elaborated in great detail on the thought process of an adolescent girl who thought about her future and how her risk-taking behavior might negatively impact her future success.

*Raymond: Some people think about their future; when I do such a thing now, what are the likely outcomes in the future? So, they think twice before they act. Some children have barely anything, but they decide to stick to their education until they complete it. This is because they look way back at their home and acknowledge that they are very poor people and say to themselves, “If I treat myself luxuriously and get this and that, I may contract some diseases or become pregnant, things of that kind, which may deter me from completing my studies.”*

Other caregivers also emphasized that those who valued their education and prioritized it would not engage in sexual risk-taking, as illustrated by Brenda, “*Some like their education a lot. She can think about the guardian’s school fees and then says no, let me leave these things and finish my education instead.”*

Relatedly, some caregivers discussed that those who “fail to have a future goal” would be at risk of sexual risk-taking. Emmanuel also pointed out that some girls might be reluctant to pursue their education because they might be older than their peers and feel like they do not belong.

*Emmanuel: Some think that they are not able to continue with their studies or that they are too old to be in school with these ones. You tell them that schooling is not attached to age, yet some sit their primary seven at twenty years old.*

***Religiosity.*** The second most frequently mentioned protective factor was religiosity. Caregivers expressed that “trusting God” or being “God-fearing” would prevent adolescent girls from being involved in sexual risk-taking. Shakirah’s account illustrated the perspective that a child who grew up in a household that believed in God would follow His teachings, including not engaging in sex.

*Shakirah: What prevents them from engaging themselves number one is the power of God because God told us not to defile our bodies; they should be holy, go at the right time. So, if the home is Godly and she is Godly and she sees God at home, it prevents her from engaging in sexual risk behaviors when she is young.*

Talia reiterated a similar point and added that it was not about which religion one belonged to as long as one followed its teachings.

*Talia: Some of the children are religious, and that prevents them. If a child is religious, it is very difficult to get involved.… The religion does not matter as long as the child is keen on praying. Whenever you pray in your religion, you get good messages. If they learn and follow, those children are protected. Religion shapes a child.*

Two participants, Olivia and Bridget, mentioned that “*lack of God’s character and counsel*” and *“not being God fearing*”, respectively, would “*make an adolescent girl engage in sexual risk behaviors*” (Bridget).

***Temperament.*** Another individual factor that was mentioned as often as religiosity was the adolescent girls’ “*nature*”. This was discussed mostly as a protective factor, but also as a risk factor by some caregivers. Dianah gave an example from her lived experience where she left home and lived with different men and shared that other adolescent girls might choose to protect themselves because of their “nature”.

*Dianah: It is because of nature with some people. Now, I left home when I was seeing people and living with them, but I had my nature. Even where we are now, there are a lot of married women who leave their homes and roam about, even my friends. But there is someone who naturally doesn’t even like to hear that. Now, that is their nature when they don’t want such. One says, “Let me protect myself. I will not do this.”*

Other caregivers used the term “*heart*” and shared that those with a “*strong heart*” would choose to counsel themselves not to engage in sexual risk-taking, which she thought was more effective than any other person counseling them, as illustrated by Joanna:

*Joanna: It’s all in the heart; people have different hearts. If you have a weak heart, you might get problems, but if you have a strong heart, you get peace, so you will not be greedy. You could counsel her as a parent or any other person, and she does not respond, but if she counsels herself, she might not engage in sexual risk behaviors.*

Similarly, Emily shared that those who have the “heart” would endure even when other people tried to influence them otherwise, “*For children who don’t engage in sexual risk behavior, it is their heart, so that even when they are influenced, they cannot engage in it but reject it highly while remembering that education is the focus.”*

Farooq called it “conscience,” which allowed adolescent girls to distinguish between “the good or bad” and act accordingly, *“Their conscience may tell them that the thing they’re going to engage in is good or bad. When they have that sense of distinguishing between wrong and right, in those circumstances.”*

Conversely, some caregivers, like Raymond, used terms like “ill-mannered” or “stubbornness” to refer to adolescent girls who were reluctant to listen to advice and resist “temptations”.

*Raymond: Some of these girls are ill-mannered; they never take their parents’ advice at home, and most of the time, even if they are advised, they never pick anything. So, when a person is ill-mannered, they may also fall victim to such temptations.*

***Concern about negative health outcomes.*** Some caregivers discussed fear of disease and pregnancy as a reason why adolescent girls would refrain from engaging in sexual risk-taking. Hamidah shared that those who were aware of the health risks associated with it would not engage in unprotected sex*, “When a child is aware that when she gets involved, she will be infected with HIV and other diseases and also knows that her life will be disorganized when she gets pregnant and drops out of school.”*

Daisy also mentioned that the knowledge that one might acquire HIV or get pregnant made them decide to restrict themselves, “*When someone decides with their own heart that I will not go there. If I go there, I will contract HIV/AIDS, or I will get pregnant. Things of that kind...She says, “I will not go there”. She restricts herself.”*

Rachael shared that those informed by their parents about the risks involved might decide to abstain until they get married, “*Sometimes they talk to their parents and learn that if you engage in sexual activities, you can get sexually transmitted diseases or get pregnant. Then, some decide to abstain until marriage.”*

***Puberty.*** Caregivers identified adolescence as a developmental stage during which adolescent girls would naturally be inclined to engage in sex and associated risk-taking behaviors, perceived as “urge” or “desires”. Peace shared that when a girl reaches a certain age, she may get “desires” that might make her engage in unprotected sex.

*Peace: Also, when she reaches the age, just like her current age, and maybe also the body reaches the time and gets desires, that’s why sometimes they just give in without first getting any protective method, and they have unprotected sex.*

Similar to Teopista, who thought that one may *“be tempted to go with what the body has decided for her”,* Emily acknowledged that it was *“science”* and that girls should receive sexual and reproductive health education during this phase.

*Emily: I tell you it is science. That is why I said it is necessary to begin teaching girls when they start their menstrual period. Because they taught us in science class that when a girl starts her period, her body changes and she feels different, and it attracts her to [sexual activities].*

Naomi called it being *“under the influence of adolescence”,* a period during which they would believe *“what they are doing is always right”.* Two caregivers talked about situations where adolescent girls do not go through those changes as a protective factor. Peace characterized “dormant nerves” as a protective factor.

*Peace: Some people say that when a girl reaches that stage of engaging in relationships, sometimes her nerves are dormant, and I don’t know whether it’s a disease or something else. I am not sure because every person was created differently by God, and their bodies are also different from others.*

Similarly, Andrew shared that depending on their “temperature”, adolescent girls would decide whether they would engage in sexual activities or not.

*Andrew: It depends on the nature of their bodies. If they are sexually active, they will engage in them, but if they are sexually dormant, they ignore them. They vary. If their temperature is high, you will find them getting involved in love affairs, and if their temperature is low, they won’t involve themselves in those affairs.*

### Proximal

***Family.*** Family-level factors were discussed both as protective and risk factors and included parental supervision, parental counseling, family relations, and parents engaging in sex.

**1.** ***Parental supervision.*** Parental supervision, specifically regarding girls’ “*movement,*” was discussed as a family-level factor that influenced girls’ decision to engage in sexual risk-taking. Tracy elaborated on the need for girls to be accompanied by their caregivers when going to places to protect them from peers who might be a bad influence.

*Tracy: When she asks you to go somewhere, don’t allow her. But when you are going somewhere, you can take her with you, and then she associates with peers she meets there, and they discuss their stories so that you understand that she is on the right path, and when you are leaving, you go back together. You shouldn’t allow her to participate with her peers in your absence, since they meet many other peers who behave differently.*

Isaac talked about the importance of having a strict curfew time, as well as being strict with boys who might come visit.

*Isaac: It’s all about limiting their movement and giving them conditions that whenever it clocks to 6:30 pm, I expect you to have reached home since you leave school at 6 pm sharp…. For these boys who visit, once they come, just welcome them and ask what the reason is for their visit. If they do that the following day and treat the situation just like the other day, now he learns that someone’s home isn’t easy to joke with.*

Relatedly, Raymond talked about parents not supervising their daughters adequately as a risk factor.

*Raymond: First of all, I will shift the blame to some parents who do not fulfill their responsibility, whereby they leave their children with much liberty, not caring about them, and they get access to go out however they want.*

Lilian echoed similar sentiments where a caregiver giving their daughter too much freedom would allow her to engage in sexual risk-taking. She argued that if stricter supervision were in place, she would refrain from doing so.

*Lilian: You give her a lot of freedom to attend, go to see friends, I think this would push her into it but if you refuse her certain things she may be stopped. If she doesn’t go parties for a year, she rarely goes to town once or twice in a month all this will stop her movements…. So, this strictness with the children could help.*

For Shakirah, caregiver supervision also needed to include what an adolescent girl should and should not be allowed to wear.

*Shakirah: Dressing up indecently, if you leave her to dress in skimpy dresses, someone can tell her a word, and she has never heard about it in her thoughts, so that can attract her because of the dress code, yet she has not yet reached the time to dress up like that.*

**2.** ***Counseling from parents.*** Counseling provided by the parents/guardians was another family-level factor caregivers identified as a critical factor contributing to adolescent girls’ decision-making about sexual risk-taking. Rose pointed out that caregivers should make their daughters aware of *“the danger of getting involved in love affairs, for example, informing them that they can get pregnant and diseases,*” so that they could be aware. Emmanuel shared that counseling and guidance from parents should start at an early age so that they can learn to differentiate the good from the bad and make responsible decisions when the time comes.

*Emmanuel: It is very important when the child is still young for the parent to counsel and guide before they get to the stage when they have to decide on their own, because we keep her account, she is not yet of age to decide on her own. When she gets to know what is bad and good, then the parent can release the child into the world to see what is good and bad.*

Relatedly, Atwaan discussed the risk of not counseling adolescent girls and the importance of talking about risks associated with engaging in sexual risk-taking.

*Atwaan: [Sexual risk-taking happens] when the parents take the responsibility of counseling their children carelessly, like not telling them the consequences of having sex when they meet with adolescent boys. However, when you sit and talk, you counsel her and tell her the reality that when you go there and have sex, you will get pregnant, you will lose this.*

**3.** ***Family relations.*** Family relations were a factor largely discussed as a risk factor, but also mentioned as a protective factor by some caregivers. Phoebe and Joanna identified *“child maltreatment at home*” as a reason for sexual risk-taking. Brenda added that domestic violence could lead to sexual risk behaviors among adolescent girls.

*Brenda: The other thing is domestic violence in the family where she lives. Let’s say when mom and dad are quarreling and fighting, the children get fed up, and when they are grown up. Sometimes, dad can finish beating up the mom and turn to the child, which makes them fed up.*

In addition to domestic violence, parental drinking problems were brought up by Shakirah

*Shakirah: The other reason they go for sexual risk behaviors is conflicts in the home. If parents behave badly at home without acknowledging the presence of children, they hurt each other, or have drinking problems, all this can result in the child engaging in sexual risk behaviors.*

Additionally, two caregivers, Sheila and Helena, mentioned fear of caregivers as a protective factor against sexual risk-taking. Specifically, Helena shared that if the adolescent girl thought “*when I go to a boyfriend, my mother will feel bad and get angry with me”,* that would deter her from sexual risk-taking behaviors. Similarly, Sheila mentioned that when adolescent girls knew they would face consequences from their caregivers, they would be less likely to engage in those behaviors.

*Sheila: That is very important if the child fears her parents. It prevents her from being tempted. But if she stops fearing her parents, there is no way of preventing her. If she fears her parents, she can’t engage in sexual risk behaviors because if her parents know, she will be beaten or given hard punishments.*

A few caregivers mentioned the positive child-caregiver relationship as a protective factor, as illustrated by Casiim, *“If she has a good relationship with her parents and they care about her, this can keep that child safe.”* Similarly, Olivia mentioned that *“affectionate relationships with one’s daughter”* would keep them safe.

**4. *Caregiver sexual behavior.*** Some caregivers talked about parents’ sexual activities at home as a risk factor. They argued that caregivers engaging in sex might encourage adolescent girls to experiment with sex, as illustrated by Alice:

*Alice: Sometimes it’s caused by some parents themselves. Parents sometimes engage in sexual activities at home, not considering their grown-up children. This can also force the young girls to engage in this activity, but they are influenced by their parents.*

Lilian shared that caregivers should be particularly careful in households that do not have sufficient privacy.

*Lilian: Also, you could have a small house at home, and your parents sleep near the children so if a child is fifteen years old, don’t sleep near her she may listen to you (having sex) and want to practice so if the house is small let them sleep in the room adjacent to the kitchen or boy’s equators, because this could tempt the child to do what she would not have done quickly.*

Conversely, Alice mentioned that parents’ responsible behavior around sex would protect their adolescent girls from engaging in sex prematurely and carelessly.

*Alice: Also, some parents behave responsibly regarding sexual behaviors, taking into consideration their grown-up children. This will help to protect the child. I mean, if the parents do not practice this act when their children are hearing or seeing, it will never persuade them to engage in such activities.*

***Peers.*** Peers were mainly discussed in the context of negative peer influence, though some caregivers also mentioned the potential protective role of peers. Bridget shared that *“bad groups can make her attracted to things that come easily and also get attracted to sexual risk behaviors before her age.”* Similarly, Rose suggested that being part of peer groups, *“advising themselves that when you go to men, you can get what you want,”* may push adolescent girls into sexual risk-taking. Monica thought that when adolescent girls see their girlfriends who *“are well off with money, they can persuade her to get a boyfriend who can provide for her, and then she gets tempted.”*

When asked about what prevents adolescent girls from engaging in sexual risk-taking, Racheal mentioned staying away from peer groups and discussed the risks associated with spending time in “bad” peer groups:

*Racheal: I think they stay away from bad peer groups, or sometimes they don’t think about it. At times when girls have those peer groups, they talk about different experiences and they encourage their friend who has never done it before to go ahead and try, since nothing can happen to her.*

Joel emphasized that positive peer relations would indeed be critical to preventing sexual risk-taking:

***Joel:*** *What helps such girls most is the friends they associate with. These friends may be humble, obedient, and well-nurtured, whereby if their friend is trying to acquire a weird behavior, they will try to bring her back to discipline.*

Helena shared that in some cases, bad examples could serve as a deterring factor for adolescent girls, *“When she is at school, she admires and says, ‘If I were like the one who never went to school, I would also be like that.’ So, she goes back to school.”*

### DISTAL

***Material needs.*** Material needs and whether one could or could not satisfy those needs were the most widely mentioned factors within the distal level as well as across all levels. Some caregivers mentioned basic needs not being satisfied by the caregivers. Maureen talked about families not being able to afford shoes for their daughters, in which case, they would look for alternatives to secure the money.

*Maureen: Poverty can make her get involved in sex behavior. She can tell you, “Mother, I do not have shoes.,” and you tell her, “I do not have money”. When you tell her that you do not have money and there is someone who can give her five thousand shillings, that can make her get involved.*

Fortunate also mentioned poverty as a reason for girls to engage in sexual risk-taking. She acknowledged that adolescent girls needed both their basic needs to be met as well as their needs for “*good things*”. She argued that girls would turn to boys when those needs could not be met by caregivers.

*Fortunate: Poverty in the family! Children need good things, but when they ask for them, parents can’t afford or provide them. The other is food; when have little food at home, children may be tempted to engage in sexual risk behaviors. A child may ask you for such good things as a beautiful dress, which you can’t afford, yet outside, there could be a boy who has promised her that dress. We parents with many responsibilities have to divide up the money we have into different domestic needs and savings.*

Kevin also illustrated both basic needs, specifically sanitary pads, and other things that adolescent girls might want because their peers had them, and how men could take advantage of the situation.

*Kevin: For example, she goes to school when her friends have what to eat at break time, but she does not. When a boda boda rider gives her ten thousand shillings a week, she will be happy, but it is not free money. Another thing that disturbs girls is getting pads. When they use pieces of cloth, they are smelling, yet they do not have money to buy pads. A man will give her five thousand shillings to get the pads and such things, put the girls in temptation.... You find that a child wants earrings, I know that most girls wear small earrings, but one may choose the big ones, buying pancakes, chapatti, a rolex (a special type of breakfast food), and the like. A boy can fetch about ten jerry cans of water and earn money to take to his girlfriend at school to buy pancakes, but she has to pay!*

Other caregivers also talked about adolescent girls “admiring” things, but not being able to afford them, as the reason for engaging in sexual risk-taking, as illustrated by Joel:

*Joel: Another thing is that young girls admire a lot of things, and yet parents cannot afford to provide all the requirements, so they end up being deceived by men to engage in a sexual relationship by the use of money and small gifts, so they end up going for the practice.*

Tracy suggested that even when a caregiver was not able to afford her daughter’s needs right away, talking to her about when it might be possible might prevent her from engaging in sexual risk-taking.

*Tracy: When she asks you for new shoes and you just abuse her when there’s a boy who promised her 10,000/= and the shoes coats 10,000/ = , do you see how she brings the shoe when you aren’t the one who bought it or even her father? But when you ask her to be patient you will give her the money after selling something let’s say chicken, even when you haven’t given her at that time, she becomes confident that mother will give me the money but when you don’t respond to her desires, the boy will give her the money.*

Relatedly, some caregivers identified caregivers’ ability to meet their daughters’ material and basic needs as a protective factor. Esther shared, *“When you provide well their basic needs and they become satisfied in their heart, [they don’t engage in sexual risk taking.]”* When asked about what would prevent a girl from engaging in sexual risk-taking, Brian said, *“If she gets everything she needs at home, for example, clothing.”*

***Program counseling.*** Though less prevalent, caregivers also discussed the availability –and lack thereof- of program counseling as a factor that influences adolescent girls’ decision to engage in sexual risk behaviors.

Lilian discussed how a counseling session with a health worker might be more effective than caregiver counseling and might encourage an adolescent girl to stay away from negative peer influence.

*Lilian: If a child gets a chance to sit with a health worker, and they explain and counsel them, she may cut off some friends. But if a child has no one to counsel her, a parent will talk, but this child will not listen. When a mother talks, she tells her that you do things the old way. If this child sits with someone from an organization, a health worker, she may listen and tell herself I think what this woman was telling me is bad, let me cut off friends or stop doing what I wanted to do.*

Peace talked about how girls might be able to refer back to what they learned in their counseling sessions if they were exposed to any.

*Peace: Sometimes they make a flashback on their background and refer to lessons that when we were learning madam so and so taught about this that you have to be patient about the goal and you first complete then you begin to engage in relationships, so many of them they get the chance from the sessions they have attended and those who have learned and take it, she reflects when she is going to do that thing, that’s how many are saved from due to counselling services you give them….*

## Discussion

In this study, we explored caregiver perspectives on factors contributing to adolescent girls’ decision-making about engaging in sexual risk-taking behaviors in Uganda, a country heavily impacted by HIV. At the personal level, future goals, religiosity, temperament, and puberty were discussed as factors that could serve as protective or risk factors. Concerns about negative health consequences were identified as a personal-level protective factor. At the proximal level, family-related factors, including parental supervision, parental counseling, family relations, and parents’ engagement in sex, were discussed both in the context of protective and risk factors. Peer-related factors included positive peer role models and avoiding bad peer groups as protective factors and negative peer influence as risk factors. At the distal level, material needs and program counseling were identified.

The most commonly discussed factor across all three levels was material needs, primarily as a risk factor. Caregivers believed that if adolescent girls were not able to meet their basic needs and/or afford other “*good things*”, such as a dress or a snack, that their friends might be able to, they would be more likely to engage in sexual risk-taking. Conversely, they thought that caregivers’ commitment to providing for their daughters would serve as a protective factor. Our results support the existing evidence that identifies poverty as a risk factor for sexual risk-taking among adolescent girls in SSA [37,38]. For instance, Nash et al.’s study [38] found that poverty was a push factor for transactional sex among adolescent girls in Malawi. Our results from this same Suubi4Her study that examined adolescent girls’ perspectives on why their female peers engaged in sexual risk-taking in Uganda also identified poverty as the most common risk factor [39]. Some studies have associated the desire for “luxury” items with peer influence, as having these items would ensure acceptance or social status among their peers [19,40,41]. While acknowledging the importance of peer influence in the desire to acquire these luxury items, we argue that engaging in sexual risk-taking as a means to secure them results from poverty, as adolescent girls would be less likely to engage in these behaviors if their families were able to afford these items [39].

At the distal level, program counseling was mentioned less frequently and primarily as a protective factor. Caregivers believed that those who had participated in sessions offered by different programs on risks associated with unprotected sex would be less likely to engage in sexual risk-taking. These results suggest that caregivers may be supportive of their daughters attending sexual health education sessions as a preventive measure. Interestingly, program counseling did not emerge as a factor in our qualitative interviews with adolescent girls in the same study [39]. Yet, several studies indicate the protective role of sexual health education in reducing sexual risk-taking, including delayed sexual debut, increased condom use, increased knowledge of STIs, and improved condom negotiation and contraceptive use [42–45].

At the proximal level, family-level factors included parental supervision, parental counseling, family relations, and parents’ engagement in sex. Caregivers believed that if caregivers provided close supervision on their daughters’ whereabouts and did not allow for “too much freedom”, adolescent girls would be less likely to engage in sexual risk-taking. Similarly, they emphasized the critical role of caregiver communication with their daughters about sexual health and risks associated with unprotected sex. Finally, the quality of the caregiver-child relationship was discussed, where parental warmth and responsiveness were identified as protective factors and domestic violence/maltreatment and lack of parental warmth as risk factors. These results align with the existing literature that identifies parental monitoring, family communication about sex, and positive family relations as protective factors against sexual risk-taking, including transactional sex, older sexual partners, and early sexual debut [6,12,23,46–48]. Specifically, our qualitative results from adolescent girls who participated in the Suubi4Her study showed that the ability to talk to their caregivers about sex and the emotional connection they had with them prevented them from engaging in sexual risk-taking [39]. Similarly, adolescent girls with more supportive family relationships were more likely to report lower levels of sexual risk-taking intentions in Uganda [48]. Interestingly, some caregivers in this study emphasized the role that caregiver sexual activities at home might play in adolescent girls’ decision-making. This was not identified as a factor by adolescent girls who participated in the Suubi4Her study [39] and has not been widely discussed in the literature. Hence, further research is needed to explore its potential role.

Caregivers also discussed the role of peers, another proximal factor, in adolescent girls’ engagement in sexual risk-taking. Contrary to the results from the adolescent girls in the study [39], caregivers mostly discussed the negative influence of peers. Some caregivers mentioned that seeing “bad” examples of what might happen and surrounding oneself with good friends would serve as protective factors, results similar to the ones from the adolescent girls in the study [39]. The relationship between peer influence and sexual risk-taking has been well-studied in SSA [49,50]. A systematic review of studies conducted in SSA identified that peer influence was an important factor contributing to adolescent pregnancy [50]. Another systematic review and meta-analysis of the determinants of risky sexual practices in Ethiopia identified peer pressure as a factor positively associated with risky sexual practices [51]. Similarly, a qualitative study of youth (13–14 years old) in South Africa showed that wanting to belong to a peer group undermined their ability not to engage in sexual risk-taking [52]. A study conducted among adolescents living with HIV found a significant relationship between peer pressure and more approving attitudes toward sexual risk-taking [53]. Conversely, the quantitative results from the baseline data in the Suubi4Her study found no significant relationships between peer pressure (direct or indirect) and sexual risk-taking [54]. Hence, further research is needed to examine the relationship between the two.

At the personal level, having future goals was identified as an important factor. Caregivers believed that adolescent girls who had future goals would be more cognizant of how sexual risk-taking might undermine their plans and would be reluctant to engage in such behaviors compared to those who did not have any plans for the future. These results overlap with those we found among adolescent girls in the Suubi4Her study [39]. The qualitative interviews with the adolescent girls revealed that they perceived sexual risk-taking as a threat to their potential to attain their future goals [39]. Studies in the US documented the relationship between future orientation and behavioral outcomes during adolescence [55,56]. However, more research is needed to understand the relationship between future orientation and sexual risk-taking, including in SSA. Our qualitative results provide insights into the potential protective role of future orientation and suggest that interventions that promote future goals may reduce sexual risk-taking.

Adolescent girls’ religiosity was the second most common factor discussed under the personal level factors. Contrary to our qualitative study with the adolescent girls [39], caregivers more frequently identified religiosity as a factor. They believed that those who were religious would be more likely to refrain from sexual risk-taking. These results align with the existing evidence that documents the protective role of religiosity against sexual risk behaviors [57–59]. However, [60] caution against the significant but small protective role of religion in young people’s sexual risk behaviors based on their meta-analytic review of 22 studies, including two in SSA.

Girls’ temperament or nature was primarily discussed as a protective factor. Caregivers mentioned that if an adolescent girl had a “strong heart” or conscience, they would resist sexual risk-taking as they would be able to differentiate right from wrong, as opposed to those who did not have a strong heart. In addition, puberty and changes associated with it were identified as a risk factor. Other studies have also documented the relationship between impulsivity during adolescence and sexual risk-taking. For instance, a meta-analysis of 40 studies examining the relations between adolescent sensation seeking and risky sexual behaviors found a significant overall mean effect size between the two, with a stronger relationship among females compared to males [61]. Another meta-analytic review that examined the relationship between impulsivity and risky sexual behavior found a significant but small relationship between the two, with similar effects across different risky sexual behaviors and impulsivity traits [62]. However, it has been demonstrated that not all adolescents take risks [63]. Some studies suggested that those with higher reward drive, an individual’s tendency to pursue rewards and/or seek novel experiences [64], may be more susceptible to risk-taking [63].

Finally, caregivers shared that those concerned about negative health consequences would be more likely to abstain from risky sexual behaviors. Studies in SSA have documented the relationship between sexual health knowledge and sexual risk-taking [41,65]. For instance, a qualitative study conducted by Ssewanyana and colleagues [41] found that limited knowledge of risks associated with unprotected sex was a risk factor. Similarly, a study in Ethiopia found a positive association between early sex debut and poor access to reproductive information among college students [65]. The results from our qualitative study with the adolescent girls also identified concerns about negative health outcomes as a personal level factor that prevented them from engaging in sexual risk-taking [39]. A systematic review of randomized controlled trials (RCT) examining the impact of school-based interventions on promoting safe sex behaviors and reducing sexual risk behaviors among young adolescents in sub-Saharan Africa found that two studies that measured pregnancy showed significant declines in unintended pregnancies and of the five studies that measured HIV/AIDS knowledge, condom use, and attitudes toward HIV testing, four detected significant improvements in all those outcomes [12].

Our comparison of our qualitative results from caregivers reported in this study and their adolescent daughters, published elsewhere [39], provides important insights that have implications for how and when caregivers may choose to engage in conversation with their adolescent daughters about sexual risk-taking. For instance, both caregivers and their daughters acknowledge the importance of a positive family environment and open communication and counseling around sex. Both groups identified poverty as a risk factor, and relatedly, families’ ability to afford their daughters’ needs as a protective factor. Hence, caregivers would be more likely to engage in such conversations when they are not able to meet the needs. Caregivers and adolescent girls also perceived the role of peers similarly, acknowledging both the negative and positive roles they may play. Hence, when and if caregivers engage in such conversations around peers, it is likely to resonate well with their daughters. The importance of religiosity was less pronounced by adolescent girls compared to caregivers. So, overemphasis of religiosity by caregivers may not always lend itself as expected among their daughters. Stigma associated with sexual risk-taking and early pregnancy, as well as what men may do after they engage in unprotected sex, were raised as factors deterring them from engaging in sexual risk-taking by adolescent girls. However, these factors were not considered by caregivers and will likely not be included in any conversations they may have.

### Study Strengths and Limitations

The following limitations should be considered. The qualitative interviews were cross-sectional and conducted after the Suubi4Her combination intervention was completed. Hence, caregiver responses may have been impacted by their participation in the intervention, and some factors may have been more pronounced. However, our stratified sampling strategy allowed us to recruit caregivers of adolescent girls who performed lowest and highest in targeted intervention outcomes (sexual risk-taking, mental health, and savings) and hence capture a wide range of experiences. Second, we report on caregivers’ perceptions of why adolescent girls engage in sexual risk-taking and not on the factors that contribute to their daughters’ decision-making. Accordingly, we cannot conclude with certainty that these would be the same factors they consider when and if they choose to intervene to reduce their daughters’ sexual risk-taking. However, we believe that caregivers may be more reserved when talking about their daughters’ risky sexual behaviors, and social desirability may be of concern. Future qualitative studies may be conducted longitudinally before participation in and upon completion of an intervention to qualitatively explore the changes, if any, in the makeup of protective and risk factors as a result of intervention participation.

## Conclusions and implications

This qualitative study provides insights into caregiver perspectives on factors that influence adolescent girls’ decision to engage in sexual risk-taking in Uganda, a country heavily impacted by HIV and poverty. Adolescent girls and young women are identified as a priority group for HIV prevention, care and treatment, and social support interventions, as outlined in the National Strategic Plan for HIV and AIDS by the Ugandan government [66]. Hence, it is important to consider incorporating multi-level combination interventions into national HIV prevention programs and policies.

Our comparison of the perspectives of adolescent girls and their caregivers showed that while there are overlaps in what constitutes a risk or protective factor, the importance they attach to it may be different. Similarly, there were distinct factors that were brought up by caregivers versus adolescent girls. These have important implications for when and how caregivers will choose to talk about sexual risk-taking with their adolescent daughters, how receptive adolescent girls will be, and whether their concerns and needs are met. Hence, our results from this study suggest that comprehensive and tailored approaches to interventions addressing sexual risk-taking among may need to consider caregiver involvement to optimize impact. In addition, the intersecting multi-level factors documented in this study underline the importance of combination interventions that may simultaneously address several risk factors while strengthening protective factors. These interventions should target both caregivers and adolescent girls and consider incorporating family strengthening approaches, poverty reduction strategies, conversations about peer influence, and support for positive future orientation, in addition to sexual health knowledge.
